# Validity of Psychiatric Evaluation of Asylum Seekers through Telephone

**DOI:** 10.1155/2021/8856352

**Published:** 2021-02-10

**Authors:** Samuel Yang, Regina Baronia, Yasin Ibrahim

**Affiliations:** Department of Psychiatry, TTUHSC School of Medicine, 3601 4th Street Lubbock, TX 79430-8103, USA

## Abstract

The goal of the psychiatric assessment of asylum seekers is to evaluate the asylum seeker's mental health and credibility. The shortage of mental health providers trained in this particular type of evaluation makes in-person evaluation not always feasible. Telephonic interview has been occasionally utilized to fill this void. The validity of such evaluations in assessing credibility has yet to be fully established. In the case of telephonic interviews, evaluators are limited with no access to facial or body language cues that can indicate deception or honesty. We will present a case of a client evaluated via telephone that was deemed credible and eventually released to pursue asylum in the US. Assessment of credibility relied solely on cues obtained from the client's narrative, reported symptoms, and their style of interaction with the evaluator. We will highlight the findings from the client's interview that supported credibility in the case and discuss the challenges of assessing asylum seeker's credibility via telephonic interview. Telephonic evaluation of credibility can be considered a valid method despite major challenges, but psychiatric evaluators should be aware of the limitations of telephonic evaluations given the high possibility of secondary gains and deception.

## 1. Introduction

Recent events have rendered immigration and asylum-seeking topics of increasing significance. According to the United Nations High Commissioner for Refugees (UNHCR), there were 79.5 million people forcibly displaced worldwide at the end of 2019 [1]. Mental health is a vital concern with refugees as studies have shown them to have higher rates of depression and posttraumatic distress syndrome (PTSD) than the general population [2]. The role of psychiatric evaluation of asylum seekers has become increasingly important due to increased recognition of the mental health needs of immigrants and asylum seekers and the effect of their mental health status on the requirement for establishing credibility [3]. The role of the psychological evaluation is to comment on clients' credibility, identifying possible malingering and explaining how cultural and mental factors could create inconsistencies in clients' narratives [3, 4]. Medical and psychological evaluations that support the applicants' account “often make the difference between successful and unsuccessful applications” [5]. These evaluations can have a significant impact on the asylum-seeking process, doubling or tripling the chance of obtaining asylum [6].

Due to the lack of trained clinicians available and experienced in providing pro bono psychological evaluations and the isolated location of detention centers [5, 7], telemedicine has been utilized to meet the high demand. Although video conferencing is typically utilized for telemedical encounters, the vast majority of detention centers in the United States do not allow video conferencing with clients. As a result, telephonic communication has become the de facto medium to conduct psychological evaluations for asylum seekers.

Two considerations with any forensic psychiatry evaluation of an asylum applicant are credibility and the possibility of malingering [3]. Several studies have commented on several factors, like culture, language, and/or posttraumatic stress disorder, that may factor in the discrepancies and inconsistencies in the asylee's autobiographical account [3, 4, 8]. With receiving refugee status as a desirable motivator, these inconsistencies could be interpreted by the asylum applicant evaluator as malingering. Malingering and deception are very common in forensic settings and continue to be a major challenge for forensic evaluators [9]. The reliance on telephonic communication for forensic evaluation will have to address these issues.

### 1.1. Malingering Indicators in Asylum Seekers

There is a consensus that combining verbal with nonverbal cues significantly increases the accuracy of lie detection to around 80% [10, 11]. However, there is inconsistent data as to whether nonverbal cues of deception are more accurate than verbal ones [12–16].

Verbal cues obtained via content-based criteria analysis and reality monitoring increased the accuracy of detecting both lies and truths to 70% [15, 17]. Such cues include the provision of a less coherent story, less spontaneous corrections of their story, fewer reproductions of conversations, fewer admissions of forgetting certain details, slower speaking, hesitation (including more “ums” and “ers”), provision of fewer details (including perceptual details), more negative emotion words, and fewer exclusive words [16, 18–20]. Linguistic studies of verbal content were also utilized and show that liars tend to use fewer first-person singular pronouns, fewer third-person pronouns, more negative emotion words, and fewer exclusive words. These linguistic characteristics showed a mean accuracy of 67% [19]

With these considerations in mind, a question arises: is a telephonic interview sufficient to detect malingering and deception. Here, we present an asylum seeker who was evaluated psychologically via telephone, was deemed credible, and was released to seek asylum in the US, reversing an appealed negative decision. We will discuss how we reached our conclusions and provide recommendations as to how psychiatrists can enhance their ability to detect malingering/deception in such cases.

## 2. Case Report

Ms. G is a 28-year-old female who used to work as a nurse in a general hospital, with no reported past psychiatric history who presented via telephone at a detention center in Texas seeking asylum with her 10-year-old son to escape physical and sexual abuse from her partner in Central America, including threats of death if she left him.

### 2.1. The Evaluation Process

A pro bono nongovernmental organization requested the psychological evaluation and arranged for a phone Spanish interpreter. The evaluation took about 3 hours. The evaluation was not recorded. At the end of the evaluation, the client provided verbal consent to present and publish her case with proper de identification. The typical evaluation includes a comprehensive psychiatric and medical history, a detailed history of the asylee's trip, and mental status examination. The asylee's responses are judged by their content such as reporting unusual or incredible symptoms or events and consistency in history and consistency between history with the mental status.

### 2.2. History of Trauma

Ms. G reported a long history of physical, emotional, and sexual abuse from her partner. She explained that he would easily get angry and on an occasion, he “held me by the neck, punched me, and scratched me, pushed me on the floor.” Initially, this occurred about once a month, but the abuse became more frequent over the past two years. She stated that his aggression was mostly related to his abuse of marijuana and cocaine. She added, “He used to force me to go with him to buy marijuana as this would make him look less suspicious”.

Eventually, Ms. G fled to the US. Luckily, her trip to the US border was void of any physical or sexual or emotional trauma. In May 2018, she and her son were arrested by ICE (Immigration and Customs Enforcement) and were taken to a detention center in Texas where they were placed in separate cells. She was about ten meters away from her son but could not talk to him as he was sitting on the floor far back in his cell, and there were too many kids crying. She stated he was scared and crying from the severe cold in the cell. She asked the officers to allow her to move to another part of the cell to talk to and comfort him but was not allowed. She continued to listen to his loud crying for a long period.

The next morning, Ms. G watched her son being taken to another cell to be transferred to another detention center. For the next two months, Ms. G. was transferred between three different detention centers during which she had her credible interview and was denied asylum but remained in detention after her lawyer appealed the decision and requested psychological evaluation. In July 2018, she and her son were reunited in a family detention center.

Ms. G described herself as a “religious” person. Her religion helps her cope with stress. Also, she stated she likes to listen to music, exercise, and dance. She has only a few friends because she does not “trust people easily.” Past psychiatric history was unremarkable.

### 2.3. Psychological Examination

Before her trip to the US, Ms. G reported symptoms consistent with Major Depressive Disorder (MDD). She reported a sad mood for about 6 months before her trip due to chronic abuse from her partner, Mr. M, worsening over the 6 months leading up to her escape.

She developed feelings of guilt because she was “not able to please him”. She began to spend most of her time sleeping and eating, leading to weight gain which further lowered her self-esteem. She reported passive suicidal ideation stating that at times she felt like “not fighting anymore” with multiple occasions where she wished to be dead. She was about to keep going by reminding herself of her son and denied any active suicidal ideation. With frequent abuse, her feeling of guilt was eventually replaced by fear and the need to escape from Mr. M. She would often feel anger towards Mr. M and worried about her future with him. She recalled one occasion when she felt paranoid and was too afraid of him to close her eyes and sleep. Despite being able to recall certain details about her abuse, she reported difficulty remembering some important details related to her trauma. When asked to elaborate, she stated that she could recall herself yelling and being hit by Mr. M on many occasions but could not remember how it all started. She denied symptoms suggestive of posttraumatic stress disorder (PTSD) prior to her trip to the US.

Since detainment in Texas and separation from her son in May 2018, Ms. G's depressive symptoms worsened, and she eventually lost interest in speaking to anyone. Her depression peaked during the first few weeks after detention when she was being transferred between centers. She reported being traumatized by the separation from her son with symptoms consistent with PTSD. She endorsed intrusive symptoms (frequent nightmares that woke her up in the middle of the night, intense recurring memory of her son sitting on the floor crying), avoidance (attempting to distract herself from painful memories), negative alterations of mood and cognition (increased anxiety, crying spells, and difficulty remembering details related to trauma), hyperarousal (difficulty sleeping, difficulty concentrating), and social impairment (poor concentration leading to difficulty conversing with people as when it was time to respond “I don't know what to say because I was not listening”). Since detainment, she also reported feeling more anxious with heart racing and shortness of breath when thinking of what the future holds for her and her son. These thoughts made it even harder for her to sleep.

Upon receiving the negative asylum decision, Ms. G's depressive symptoms worsened although she reported feeling much better since reuniting with her son on July 22^nd^, especially as he would talk to her and try to make her laugh. This was even though her son himself had become increasingly easily agitated and was crying a lot. He also started to wet his bed about three times a week, which was not normal for him.

Ms. G described herself as a “religious” person. Her religion helps her cope with stress. In addition, she stated she likes to listen to music, exercise, and dance. She has only a few friends because she does not “trust people easily.” Past psychiatric history was unremarkable.

### 2.4. Assessment of Mental Status

Ms. G had a calm disposition throughout most of the interview. She was forthcoming and would answer questions with relevant responses. She spoke slowly, mostly in short sentences, and her voice seemed stressed and hoarse. She was emotionally reactive as she laughed briefly when asked if she was a social person. She was also heard sobbing on multiple occasions when talking about the separation from her son. She was still feeling depressed and was increasingly worried about the possibility of deportation.

### 2.5. Impression

Ms. G is experiencing an episode of PTSD consistent with the trauma endured. She reports suffering from the separation from her son during initial detainment, exacerbated by the current additional stress from possible deportation. We found no clear evidence of malingering, as she was not indiscriminately endorsing symptoms and her presentation seemed reasonable considering her narrative of trauma [21, 22].

## 3. Discussion

Current literature is generally supportive of the use of telephonic communication although more research is needed. Multiple studies have documented the effectiveness of telephonic interviews in counseling and psychotherapy [23–26], although concerns were raised regarding limitations concerning the therapeutic relationship [27]. To our knowledge, only one study has directly examined the value of telephonic interviews in providing mental assessment of patients where they were found to be almost equivalent to in-person evaluation [28].

Regarding the use of telephonic communication for psychological evaluation in forensic settings such as the case presented, there are unique considerations due to obvious secondary gains and high rates (30%) of malingering and deception [29]. Rogers et al. suggested 10 strategies that malingerers usually utilize to deceive psychological evaluators many of which can be evaluated through telephonic interviews. These strategies are classified under two themes, namely, unlikely presentations (such as rare symptoms or rare symptom combinations) and amplified presentations (such as indiscriminate symptom endorsement, high severity of symptoms, and inconsistency between observed and reported symptoms) [21, 22, 30].

Considering the first theme (unlikely presentations), Ms. G. did not report any unusual or rare symptoms. Her symptoms of depression and PTSD are typical presentations. These symptoms, triggered by severe trauma, are expected to induce similar presentations in other common situations. The combination of depressed mood, high arousal, and flashbacks are typical of PTSD. Also, she stated she felt guilty because she was “not able to please him”. This is consistent with self-depreciation associated with depression. Although she has a chronic history of significant abuse from her husband, her PTSD symptoms only started after the most recent trauma caused by forced separation from her son. This resembles cumulative trauma, a well-known phenomenon when traumatized individuals do not develop PTSD symptoms until they suffer a tipping point trauma.

Considering the second theme (amplified presentations), again, Ms. G. did not seem to be utilizing such strategies. She denied initial PTSD symptoms despite abuse history and denied active suicidal ideation even after her son was separated from her. In fact, she reported some improvement of symptoms since reuniting with her son. During the evaluation, she seemed emotionally reactive and would laugh appropriately during the interview.

The above observations lead us to believe that she was not malingering and seems credible. All of these observations were easily obtained via telephone interview.

### 3.1. Utilization of Lie Detection Techniques

In an attempt to increase the accuracy of our evaluation of Ms. G.'s credibility, we utilized findings from research on clinical cues of deception.

Considering verbal cues, Ms. M's history included multiple cues suggestive of honesty. The first is the detailed description of her emotional status when living with her husband, reporting how the initial feelings of guilt were gradually replaced by fear. She also provided a detailed description of an episode when she was too afraid of him to close her eyes and sleep. Second, she admitted to multiple positive facts such as feeling much better after being reunited with her son, being able to enjoy music and exercise, and obtaining support from her religious practice. She also laughed appropriately at certain points during the evaluation. Third, despite clear memories and flashbacks of being yelled at and hit by her husband, she admitted the inability to recall some memories of how the abuse began. Fourth, we did not appreciate marked hesitancy or slow speech during the evaluation.

### 3.2. What Is Missing?

Multiple cues for deception are nonverbal and can be inferred from observing emotional facial expressions and body language. Facial expressions, particularly negative ones such as sadness and fear, are hard to feign and can be indicators of honesty. Decreased use of hand illustrators and increased blink rate are more associated with deception. Contrary to popular belief, gaze aversion was shown to be associated with honesty rather than deception [18, 31, 32]. In telephonic evaluations, evaluators miss such cues that could be indicators for deception and are not able to monitor for inconsistencies between verbal content and facial expression or body language.

### 3.3. Conclusion

Telephonic forensic evaluation of asylum seekers may be reliable when sufficient cues of honesty are appreciated. Such cues can be inferred from the client's history, reported symptoms, and style of speech. However, if clients show hints of symptom feigning, a more comprehensive approach, involving in-person interviews and psychological tests, should be utilized.

### 3.4. Limitations and Recommendations for Future Research

We did not utilize malingering-detecting psychological tests with our client. A quick and reliable test such as the Miller Forensic Assessment of Symptoms Test (M-FAST) can help assess credibility. It takes 5-10 minutes and can be administered via telephone and has sensitivity and specificity to malingering of 0.83 and 0.85, respectively [33]. Also, future research should examine the reliability of telephonic evaluation in comparison to in-person evaluation.
